# Neonatal myelomeningocele with unilateral cleft lip and palate: a case of concurrent midline developmental defects

**DOI:** 10.11604/pamj.2025.52.185.49548

**Published:** 2025-12-24

**Authors:** Sakshi Janardhan Patil, Kavita Gomase

**Affiliations:** 1 Radhikahai Meghe Memorial College of Nursing, Datta Meghe Institute of Higher Education and Research (Deemed to be University), Sawangi (Meghe), Wardha, India

**Keywords:** Meningocele, encephalocele, amniotic band syndrome

## Image in medicine

A male neonate born at term via vaginal delivery presented at birth with visible congenital anomalies. On examination, a soft, cystic swelling was observed in the lumbosacral region, covered with a thin membrane and containing neural elements. The lesion was consistent with a diagnosis of myelomeningocele, a severe form of spina bifida resulting from defective closure of the neural tube. Additionally, the infant exhibited a complete unilateral cleft lip and palate, suggesting disrupted craniofacial morphogenesis. These coexisting midline anomalies point toward an early embryological insult affecting multiple developmental fields. Myelomeningocele is often associated with neurological deficits and hydrocephalus, which requires urgent neurosurgical closure to prevent infection and preserve function. The cleft lip and palate deformity necessitates staged surgical correction and long-term follow-up involving speech therapy, dental care, and plastic surgery. Early neuroimaging, renal ultrasound, and evaluation for other systemic anomalies are recommended. Genetic counselling should be offered to the family, especially if syndromic causes are suspected. Antenatal folic acid supplementation has been proven to significantly reduce the incidence of neural tube defects, highlighting the importance of preventive maternal health strategies. This case underlines the need for comprehensive newborn assessment and coordinated multidisciplinary management in neonates presenting with congenital anomalies.

**Figure 1:**
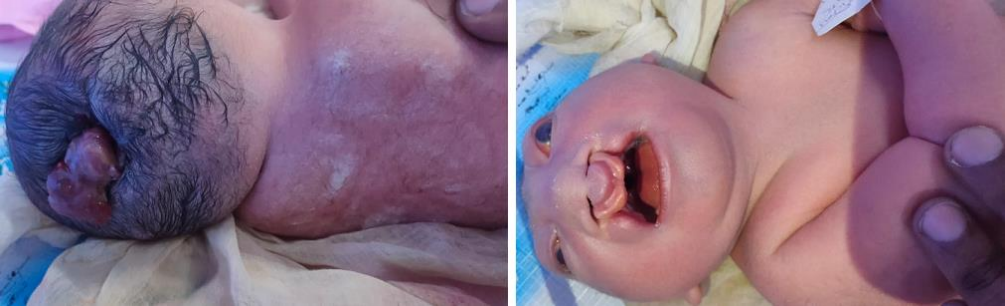
neonatal myelomeningocele with unilateral cleft lip and palate: a case of concurrent midline developmental defects

